# One-year effects of bifocal and unifocal glasses on executive functions in children with Down syndrome in a randomized controlled trial

**DOI:** 10.1038/s41598-021-96308-5

**Published:** 2021-08-19

**Authors:** Christine de Weger, F. Nienke Boonstra, Jeroen Goossens

**Affiliations:** 1 Department of Cognitive Neuroscience, Donders Institute for Brain, Cognition and Behaviour, Radboud University Medical Centre Nijmegen, P.O. Box 9101, 6500 HB Nijmegen, The Netherlands; 2 Bartiméus, Institute for the Visually Impaired, Van Renesselaan 309, 3703 AJ Zeist, The Netherlands; 3 Royal Dutch Visio, National Foundation for the Visually Impaired and Blind, Huizen, The Netherlands

**Keywords:** Neuroscience, Psychology, Health care, Medical research

## Abstract

Appropriate glasses can improve visual functioning of children with Down syndrome (DS), but it is unknown if such interventions influence their cognitive impairments. In a randomized controlled trial with 1-year follow-up. Children with DS (2–16 years) were provided either bifocal glasses (add +2.5 Dioptres; n = 50) or unifocal glasses (n = 52). Executive functions were assessed pre- and post-intervention with the task-based Minnesota Executive Function Scale (MEFS) and with questionnaires, BRIEF-P and BRIEF, parents’ and teachers’ version. Intervention effects and associations between executive functions, (near) vision and ocular alignment were analysed. Intervention improved MEFS-Total-scores in the bifocal group (*p* = 0.002; Cohen’s d = 0.60) but not in the unifocal group (*p* = 0.191; Cohen’s d = 0.24). Post-intervention, there was no intergroup difference (*p* = 0.120; Cohen’s d = 0.34). Post-intervention, higher MEFS-scores were associated with better visual acuities (crowded near *p* = 0.025; uncrowded near *p* = 0.019; distant *p* = 0.045). Pre-post changes in MEFS-scores correlated significantly with improved ocular alignment (*p* = 0.040). Exploratory analysis of the questionnaires showed improved teacher-rated BRIEF-scores in both groups (bifocals: *p* = 0.014, Cohen’s d = 1.91; unifocals: *p* = 0.022, Cohen’s d = 1.46), with no intergroup difference (*p* = 0.594; Cohen’s d = 0.23). These results demonstrate positive effects of wearing better-correcting glasses on executive functioning in children with DS, suggesting a link between their visual and executive functioning. However, the relative contributions of distant and near vision need further study.

Down syndrome (DS) is the most frequently occurring chromosomal anomaly; with an incidence of 14.6 in 10,000 live births^1,2^. The brain development in DS is slower and to a limited level compared to typically developing children^3–6^. As a result, children with DS have a varying degree of intellectual impairment with delayed cognitive and motor development^7^. The neurological deficits, as well as the ocular disorders specific for children with DS, hamper their visual acuity^8^. Reported prevalences of ocular disorders in children with DS differ between publications, but they are invariably higher than in typically developing children^5,8–21^. In 80 to 100% of the children with DS, reduced visual acuity (VA), poorer than 0.3 LogMAR, (near VA even more severely than distant VA) and reduced contrast sensitivity are found. Accommodation lags (incapacity to accurately change the shape of the ocular lens to focus the image on the retina) occur in 50 to 90% of the children with DS, strabismus (squint) in 15 to 47%, nystagmus (involuntary eye movements) in 6 to 33%, and refractive errors (inappropriate shape of the eye causing problems with focusing light accurately on the retina) are found in 40 to 90% (depending on the definition of the lower limit of refractive error) and these refraction errors are larger compared to typically developing children. The ocular disorders are mutually related and aggravate each other. For example, uncorrected refractive errors can hamper the development of visual acuity because there is no focussed image on the retina. In specific refractive errors (hyperopia), accurate accommodation can focus the image on the retina, but the accommodation is associated with convergence stimulus, which may induce strabismus which on its turn induces amblyopia (lazy eye, low visual acuity). If accommodation is poor, the attempt to accommodate can induce strabismus and, as a result of strabismus, amblyopia. Nystagmus also hampers visual acuity, but low visual acuity aggravates nystagmus. More information about the ocular disorders in children with DS and the effect of bifocal on visual acuity and strabismus in children with DS is given in our previous publications^21,22^.

Visual impairment can hamper cognitive development too. In visual impaired children without known developmental disorders, the level of visual impairment indeed correlates with deficits in cognitive development^23–27^. Cumulative debilitating consequences of early-onset visual impairment on cognitive, language and social skills are described in other studies^23,24,28^. Even children with mild to moderate visual impairment show reduced adaptive behaviour. They have more difficulties with skills that affect development and learning than normally-sighted typically developing children^28^. However, in children with DS, it is still unclear whether the visual impairments aggravate their lag in cognitive development. If this relation exists, improving their visual acuity with optimal corrections in glasses tailored to the specific ocular disorders of children with DS could support cognitive development. Children with DS might also benefit from higher visual acuities because studies performed in the last two decennia show that their visuospatial memory is relatively preserved, and better than their verbal memory^29–32^. In addition, the recent review by Lukowski et al.^33^ of studies on executive functions in children with DS underscores the relative strength in visual spatial working memory. Deficits occur in all domains of executive functions of children with DS—planning and goal directed behaviour, inhibitory control, cognitive flexibility and working memory—but children with DS perform worse on verbal working memory than on visuospatial working memory. The observed deficits in working memory are important in their own right, but its association with academic achievement in children with DS highlight its significance further^34^.

Improving visual acuity with optimal corrections in glasses tailored to the specific ocular disorders of children with DS could be a first step to support their visuospatial working memory and their cognitive development. Correcting refractive errors in the way it is done in typically developing children is not optimal for children with DS, because of their specific mixture of ocular disorders. Full correction of refractive error is required because of the lag in accommodation. Moreover, the accommodation deficit may require different correction for looking at far and near distances. In small scale studies with bifocals in children with DS, good results were obtained in accommodation accuracy^35,36^, near visual acuity and literacy skills^37,38^ through the near addition. Adyanthaya et al.^39^ studied the compliance with wearing bifocals. Of the children with bifocals, 89% were compliant whereas only 50% were compliant with unifocals. Al-Bagdady et al.^36^ (n = 40, age range 5–14 years) found that accommodation was accurate in 38 (95%) children. Nandakumar et al.^37,38^ reported that with bifocals, visual acuity improved more than 1.5 LogMAR and that 6 months later, literacy skills and school performance were improved too. However, this investigation did not include a control group, and focused only on a small group of children with DS that were pre-selected for their ability to read and write.

Triggered by these improvements in near visual acuity with bifocals^37,38^, we set up a multicentre randomized controlled trial (RCT) in the Netherlands to study the effect of bifocals in comparison to unifocals on visual functions and cognitive development. Evaluated visual functions included distance visual acuity and near visual acuity—both uncrowded (i.e., charts with a clear spacing between the symbols) and crowded (i.e., symbols printed as close together as letters in a word) acuity, accommodation accuracy, strabismus, binocularity and stereopsis. After one year, the full correction of refractive error improved distance visual acuity in both intervention groups, but bifocals led to the largest improvement in near visual acuity and better ocular alignment (fewer children with strabismus and smaller angles of strabismus)^21,22^. The improved visual acuities were a good starting point to study the association with cognitive development too. Cognitive development was assessed by testing executive functions—neurocognitive skills that serve as a foundation for early learning^40^.

The cross sectional analysis of our baseline measurements was, to the best of our knowledge, the first study to investigate the relation between lags in executive functions and visual impairments in children with DS^6^. This analysis showed a correlation between visual acuity and the level of adaptive behaviour as previously reported for visual impaired children without developmental disorders^23–28,41^. The observed correlation between visual acuity and the level of adaptive behaviour is a first indication that visual acuity might play a role in the development of executive functions in children with DS. However, at baseline, the children still wore their habitual glasses, glasses that were often not updated recently and that typically under corrected for the children’s refractive errors at the time of inclusion. Thus, in our previous study, we did not consider the effect of best-corrected visual acuity, nor did we analyse the 1-year cognitive development.

In the current paper, we assessed and compared the effects of two interventions improving visual acuity in children with DS, bifocals and unifocals, on cognitive development. We analysed the executive functions assessed in different ways and examined the relation between post-intervention visual acuity and the level of post-intervention executive functions. We hypothesized that if visual acuity influences cognitive development, intervention with glasses should have a larger effect on executive functioning after a whole year than shortly after the intervention. Second, if near visual acuity is of particular importance, we would expect a larger effect of bifocals than of unifocals. Third, we would expect a significant correlation between improvements in visual acuity and improvements in executive functions. Note, however, that distant and near visual acuity need not have the same effect on different measures of executive functions, as different measures of executive functions capture different facets of executive functioning^42,43^.

## Methods and participants

To study the difference between the effect of bifocals in the intervention group and the effect of unifocal glasses, both with full correction of refractive error, we performed a multicentre randomized controlled trial in 15 participating locations in the Netherlands. Detailed descriptions of the methods and participants of this study have been published elsewhere^6,21,22^. Here, we reproduce part of the methods, for completeness and clarity. The locations, 14 hospitals and one institute for the visually impaired, were geographically spread across the Netherlands serving rural and urban populations of diverse social economic status.

The included children from the participating institutes were randomly allocated to the two intervention groups with equal probability: bifocal group and unifocal group. The digital Web-based research data managing system, ResearchManager (2014, a web-based electronic CRF, developed by Cloud9 Health Solutions and Isala Academy in Zwolle, the Netherlands, according to GCP and GCDMP guidelines and 21 CFR part one of FDA regulations) effectuated the randomization in a permuted blocks randomization schedule, stratified by gender, age, and language development (parents report: speaking in 1 to 3 word sentences and speaking in 4 word or longer sentences). The intervention group, to which the child was assigned, was always known to the participant, the orthoptist and the investigator, because bifocal glasses are a visually prominent marker.

**Figure 1:**
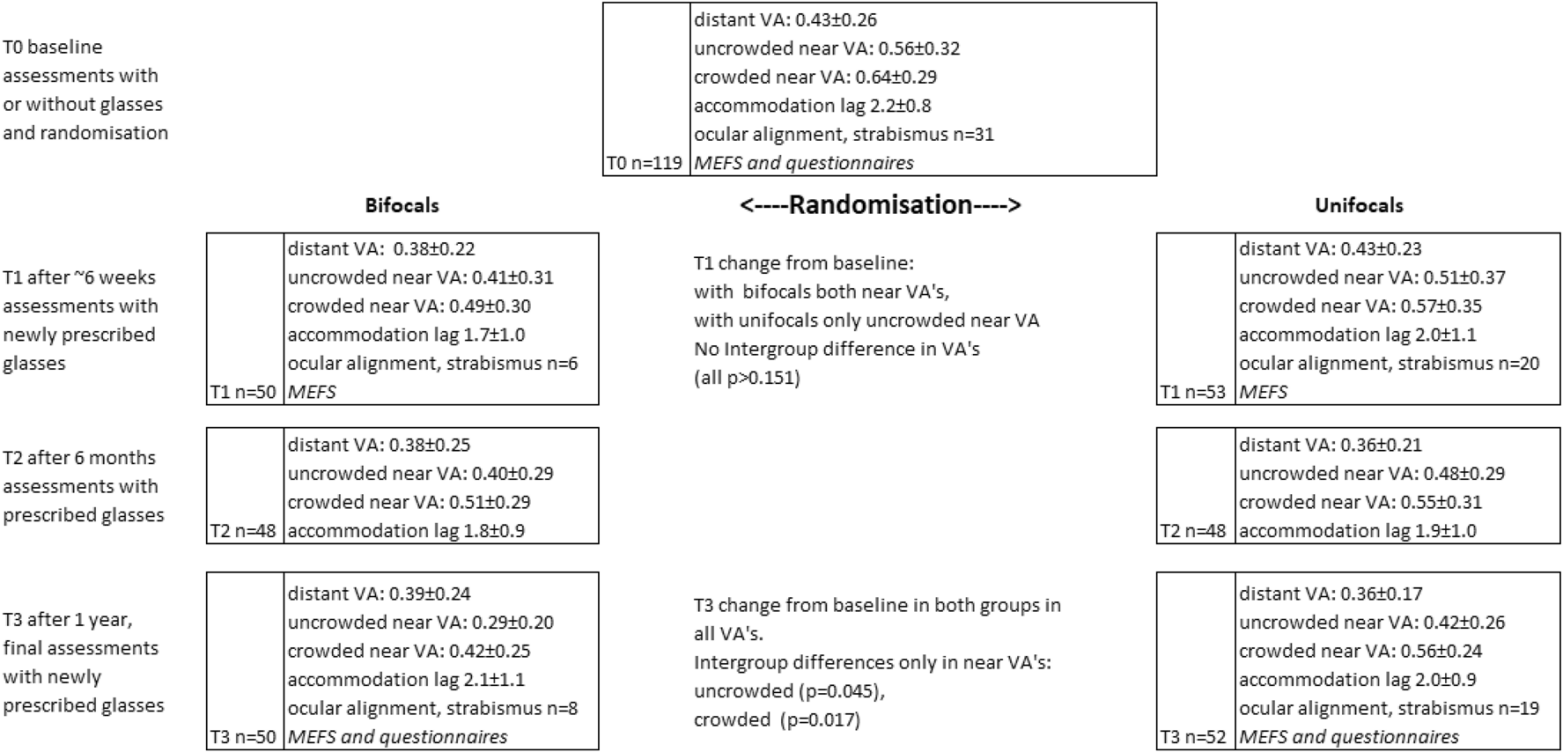
RCT time-line. Study design with the number of children tested at each point in time (n), mean visual acuities (± standard deviation. Expressed in LogMAR), with significant short-term (T1) and 1-year (T3) changes, and type of executive function assessments at baseline (T0), shortly after receiving new glasses (T1), interim check for visual functions (T2) and after 1 year (T3). Randomization was stratified by age, gender and level of verbal development. *VA* visual acuity, *MEFS* Minnesota executive function scale, a task-based executive function test.

In both groups, full correction of refractive error measured using cycloplegia was applied. The bifocal segment top of the applied longline (flat-top or D-segment) bifocals with addition +2.5 dioptres, used in the bifocal group, was placed at the pupillary centre, as used in previous studies^35,36^. In those studies, good results were achieved in improving near vision and compliance in wearing these glasses. The children were seen on four occasions, T0 (baseline), T1 ~ 6 weeks, T2 6 months after inclusion, and T3, the final assessments one year after inclusion (see Fig. 1).

This project (Clinicaltrials.gov registration number NCT02241356, registration date 16/09/2014) was approved by the Dutch Medical Ethics Committee of the Isala Hospitals (NL48288.75.14/ METC: 14.0333) and confirmed by the local ethics committees of the participating clinics. All methods were performed in accordance with relevant guidelines and regulations^21,44,45^. The sample size was calculated with G*Power 3^46^ according to results of former research^37^ on near vision before and after bifocals were used. To enlarge the inclusion number, we made some amendments to the protocol shortly after the trial commenced and these were approved again by the Medical Ethics Committee. Recruitment time, first planned for 6 months (from June to November 2015), was extended to 9 months (to February 2016), follow-up time was shortened from 1.5 year to one year (ending March 2017), and the age range originally limited from 2 to 14 years was extended to 18 years.

The current paper reports the effect of bifocals and unifocals, both with full correction of refractive error, on executive functions during one year follow up. In our previous papers, we described the baseline assessments of executive functions^6^ (Fig. 1, T0) and the effects of bifocals and unifocals on visual acuity^21^, accommodation accuracy and strabismus^22^.

### Participants

A total of 119 children with DS between 2 and 16 years old were included after written informed consent was obtained from both parents of each child, and one parent in case of single parenthood. We included children from the age of two years, the youngest age at which bifocals can be used in the appropriate way by children with DS, because visual development takes place in the first years of life and the development of strabismus may be avoided when corrections for hyperopia are used from young ages (to avoid excess of accommodation attempt, which induces convergence). At the age of two years, most children with DS can sit and perform a task at a table. This task performance induces a viewing direction which is needed to use bifocal glasses in the appropriate way. All of the included children had (1) accommodation deficit, (2) not worn bifocals before, (3) ability to respond (verbally or non-verbally) to vision tests if they were older than 5 years, and (4) were able to sit on a chair while doing a task. 104 children came back for testing with their newly prescribed glasses and were included in the longitudinal analyses described in this paper.

### Assessment procedures

Procedures for visual function examination and assessment of executive functions were protocoled. The participating orthoptists, local investigators from the 15 participating locations in the Netherlands, were trained to perform unfamiliar tests, to administer the MEFS as prescribed by Reflection Sciences, LLCTM, and to use the digital research data manager ResearchManager (2014). Additionally, each participating centre was visited by the principal investigator to review the procedures before the start and twice by an independent research monitor during the study to verify compliance of the local investigators with the research protocols.

A baseline visual function assessment was performed followed by executive functions assessment with the task-based test. At the end of the first and final visit, questionnaires were handed out to the parents to be filled out at home or were administered online by the parents or teachers, respectively.

If a child became uncooperative, testing was stopped according to the Dutch code of conduct relating to expressions of objection by people who are incapable of giving consent, minors or mentally disabled participating in medical research^45^ (Code of conduct in the Netherlands 2002, NVK Code of conduct in the Netherlands 2001). Reasons for missed data, be it a lack of cooperation or otherwise, were noted.

#### Visual functions

Visual functions were assessed at all four time points (Fig. 1). At baseline (T0) the children wore their habitual corrections or no corrections when they did not use glasses. At all 3 time points thereafter (T1, T2 and T3), the children wore their newly prescribed glasses.

##### Visual acuity

Visual acuity was assessed with verbal or non-verbal methods at distance (5 m or 3 m, according to the capacity of the child) and at near (40 cm) with symbol discrimination on visual acuity charts. Depending on the child’s capacity, we used LEA symbols^47^ or Kay pictures^48^. At 40 cm, we assessed both uncrowded (symbols with large spacing) and crowded (symbols arranged close to each other like letters in a word) near visual acuity with LEA symbols with absolute spacing^49,50^.

##### Accommodation

Accommodation accuracy was assessed at 25 cm and 16.7 cm using the ‘modified Nott method’^9,51,52^. The child looked at a small fixating object at the close distance. Meanwhile, the streak retinoscope was moved closer and further away from the child’s eyes to assess the distance of the exerted accommodation.

##### Strabismus and binocularity

In case of (nearly) straight eye position (evaluated with corneal light reflex at the beginning of the assessment) binocularity and stereopsis were assessed with one of several tests (TNO test (Lameris Ootech, Nieuwegein, The Netherlands), Titmus Fly (Stereo Optical Co., Inc., Chicago, IL), Lang Stereotest (Lang-Stereotest AG, Küsnacht, Switzerland), or positive base out 15 dioptre prism test), chosen by the orthoptist according to the developmental stage and cooperation of the child.

After that, both manifest and/or latent strabismus angles were assessed with the prism cover test at 30 cm and 5 m, the Hirschberg corneal reflex test^53^ and cover test at 30 cm and 5 m.

#### Adaptive developmental behaviour: Vineland-S

The Vineland-Screener^54,55^ (Vineland-S) was used to assess adaptive developmental behaviour at baseline (T0) only. The Vineland-S is a questionnaire for parents with 72 items. This questionnaire covers the four domains of adaptive behaviour: communication, socialization, daily living skills, and motor skills.

#### Executive functions

Executive functions (EF) were assed with a task-based method and with questionnaires. Such methods are complementary to one another^55–58^. Task-based tests are like a snapshot, a momentary assessment mostly under optimal conditions. By contrast, rating based assessments provide a score of everyday executive functioning in the daily behaviour of the children in various settings.

##### Task-based: Minnesota executive function scale (MEFS)

At T0, baseline, T1, the assessment with newly prescribed glasses after ~ 6 weeks, and at T3, the final assessments after one year, the participants themselves were tested using the task-based Minnesota Executive Function Scale^59,60^ (MEFS). The MEFS is an engaging computer card-sorting game administered on an iPad one-on-one with the child. The MEFS test, suitable for the entire calendar age range of our participants, includes 7 levels of increasing difficulty, corresponding to the Total scores of 10, 20, 30, 40, 50, 60 and 90.

The picture size of the MEFS test applied in our study is ~ 8 M which is visible for visual acuities of 1.5 LogMAR (3/100 Snellen equivalent) with an allowed viewing distance up to 15 cm.

##### Questionnaires: BRIEF-P (preschool), BRIEF parents’ and BRIEF teachers’

At T0 and T3 (i.e., at baseline and after one year), we obtained informant based ratings from the children’s parents and teachers in the Behavior Rating Inventory of Executive Function (BRIEF-P or BRIEF) questionnaires^61–66^. The parents filled in the BRIEF-P questionnaire or the parents’ version of the BRIEF depending on the calendar age of the child (in this study of children with DS, younger than 8 years or eight years and older, respectively). Teachers filled in the teachers’ version of BRIEF. These questionnaires provide an ecologically valid real-world assessment of executive functions and yield complementary information to the task-based test^56^. The BRIEF and its subscales can generally be performed in a psychometrically sound manner among school-age children with DS^56,67^. In our analyses, we only considered the raw aggregated scores across domains, the raw Global Executive Composite (GEC). Normative GEC scores in both boys and girls on the parents’ version of the BRIEF range between 72 and 216 and on the teachers’ version between 73 and 219. In BRIEF-P the normative GEC scores lie between 189 and 63 for boys and girls. Higher scores represent greater levels of executive function impairment.

### Statistical analyses

Statistical analyses were performed using the statistical package for social sciences (SPSS version 23, IBM., Chicago, IL) and the statistical software package “R” (version 3.6.2). We used mixed effects regression models with a random intercept estimated for each participant. R-code for the mixed effects regression analyses is made available on the data repository for this paper.

Adjustment for adaptive developmental age, assessed with the Vineland-S questionnaire, was not needed because calendar age and adaptive developmental age assessed with the Vineland-S questionnaire were tightly correlated (Pearson r = 0.722, *p* < 0.001 and r (partial adjusted for gender) = 0.724, *p* < 0.001).

For each intervention group, we first compared baseline MEFS, BRIEF-P and BRIEF results with post-intervention scores (pooled across T1 and T3). Then, we compared the post-intervention results between the two intervention groups. Thereafter, we analysed the relation between final visual acuities and post-intervention MEFS scores. In addition to the test statistics and confidence intervals, we report Cohen’s d as a measure of effect size. According to common convention, we interpret Cohen’s d effect sizes of 0.2, 0.5 and 0.8 as being small, medium and large, respectively.

To analyze and compare the effects of the interventions on performance in the MEFS test, we used the raw MEFS score, the Total score (further referred to as MEFS score), as opposed to its norm-referenced score, because children with DS have cognitive and motor developmental lags and their development is heterogeneous. We ran one mixed effects model on the MEFS scores obtained at T0, T1 and T3 with gender and age at T0 (in months) as covariates. As the post-intervention (T1 and T3) MEFS scores were not significantly different between the T1 and T3, we pooled the data from these two time points to maximize statistical power.

Unfortunately, there were too many missing data for each of the baseline visual acuity measures to analyze the effect of intervention-related visual acuity changes on the change in MEFS score. To make best use of the data, we instead quantified the relation between visual acuity (i.e., visual acuity with newly prescribed glasses) and post-intervention MEFS scores. We only considered the data of visits T1 and T3. The analysis consisted of several steps. We first modelled the MEFS scores as a function of gender, age and intervention using the data from subjects with no missing values. From the resulting mixed effects model, we then calculated adjusted MEFS scores, i.e., MEFS scores adjusted for gender, age and intervention for the participants with MEFS scores at T1 and/or T3. Then, we ran three separate mixed effects models for the adjusted MEFS scores with crowded near visual acuity, uncrowded near visual acuity and distant visual acuity as fixed effects, respectively. The missing data of near visual acuities and distant visual acuity would otherwise introduce changes in the coefficients for gender, age and intervention between these three analyses.

We also tested the association between the changes in ocular alignment and changes in MEFS scores between T0 and T1 by applying the Spearman rank-correlation test. In this analysis, only children with MEFS scores available at both T0 and T1 were included.

To analyse the results of the BRIEF-P and BRIEF questionnaires, we used the raw GEC scores (Global Executive Composit, i.e., the composite scores of all scales). We did not convert these scores to age-adjusted ‘total scores’ for typically developing children (as described in the manuals of the questionnaires) because children with DS have motor and cognitive delays.

Because questionnaire data were often missing, we had to limit our analyses of the BRIEF-P and the two versions of the BRIEF to an exploratory analysis of the complete cases. A paired t-test was used to analyze the differences between baseline scores and final scores. An unpaired t-test was used to compare these differences between intervention groups. We calculated the lags of the children with DS compared to norm scores given in the manual of the questionnaires, i.e., score of the child with DS minus age-matched norm score, at one year follow-up (T3). We compared these developmental lags at final assessments to the lags found at baseline^6^. For completeness, and to be aware of possible biasing factors, we compared the group of children included in these analyses to the group of children excluded because of missing data.

## Results

At baseline, the bifocal and the unifocal group showed no statistically significant differences in calendar age, adaptive developmental age, uncrowded and crowded near visual acuities and distant visual acuity, accommodation accuracy, strength of habitual glasses, ocular alignment and executive functions as assessed with the MEFS and BRIEF-P or BRIEF^6,21,22^.

### MEFS

The MEFS was successfully administered in 86 (83%) participants at baseline (T0), in 82 (79%) participants at T1 when the children just started to use their new glasses, and in 94 (90%) participants at the final assessments (T3). MEFS scores were missing for various reasons. Either the child was too young or it did not understand the test (2, 5, 3 times at T0, T1 and T3, respectively), or the child was uncooperative (4, 5, 0 times respectively) or there were technical problems with the iPad (1, 5, 3 times respectively). For the remaining cases, no cause was described. In our analyses, only participants with baseline scores who had a follow-up score at T1 and/ or T3 were included (bifocals n = 41; unifocals n = 44).

#### Effect of the interventions on MEFS total scores

**Figure 2:**
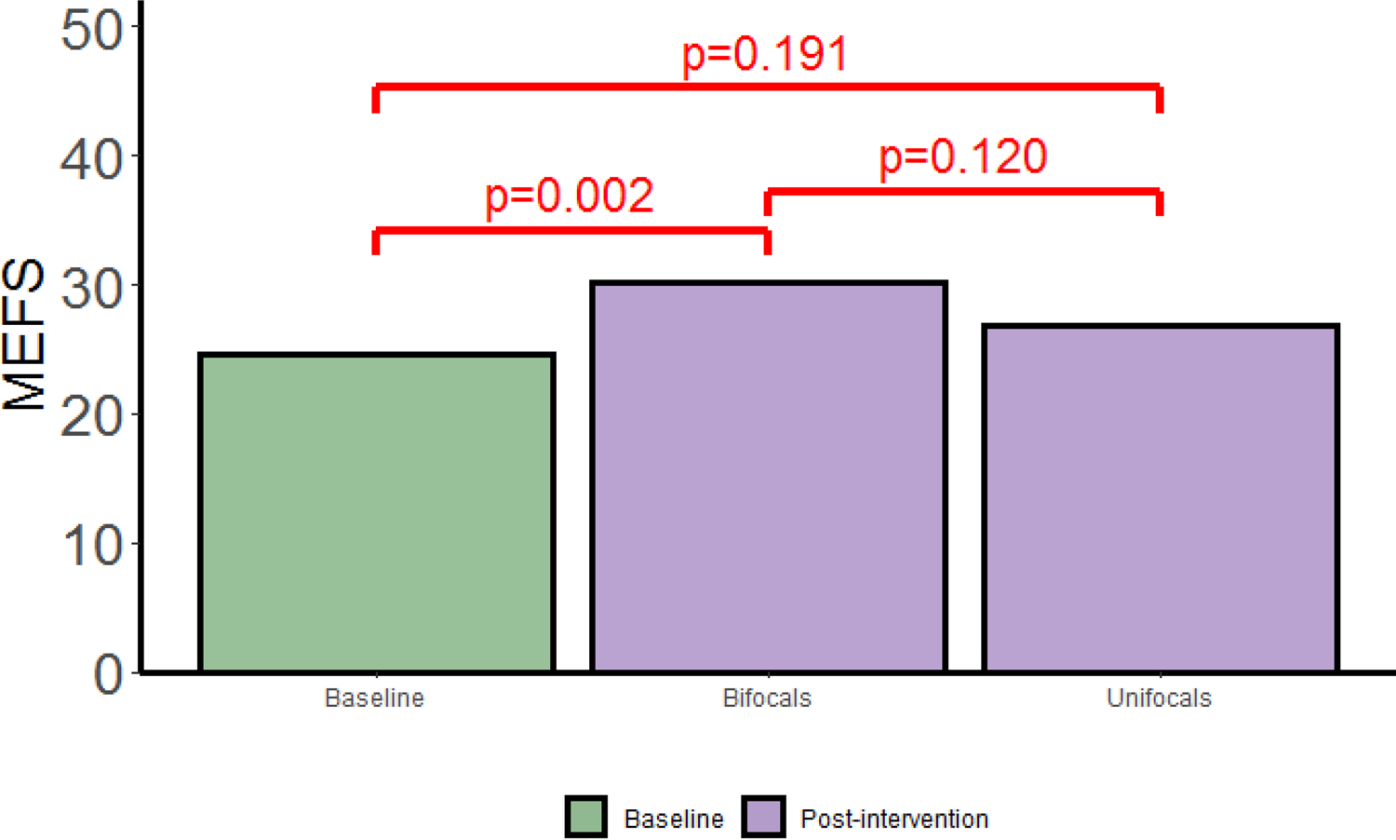
Effect of the interventions on MEFS. Note the significant post-intervention improvement of MEFS scores in the bifocal group. The intervention had no significant effect on the MEFS scores in the unifocal group. Post-intervention values (pooled across T1 and T3) were not significantly different between the two groups.

Post intervention MEFS score were correlated with age (See supplementary Table S2 online) as they were at baseline^6^. Therefore, to investigate the effect of the treatments, we took into account gender and calendar age as possible confounding factors. We ran a mixed effects model for the effect of treatment on MEFS scores adjusted for gender and calendar age at baseline across all three time points at which MEFS scores were collected (T0, T1 and T3). In these analyses, the follow-up scores were pooled across T1 and T3 since there were no significant differences between T1 and T3 (t(77) = − 1.459, *p* = 0.149; Supplementary Fig. S1 online and Table S1 online). The results are shown in Fig. 2.

The mean MEFS score at baseline adjusted for gender and calendar age was 22.4 points (95% CI: 15.0, 29.7). On the post-intervention visits, the mean MEFS score was increased to 28.0 points (95% CI: 20.5, 35.5) in the bifocal group, and to 24.6 points (95% CI: 17.1, 32.1) in the unifocal group. In the bifocal group, the medium-effect difference in mean MEFS scores between the post-intervention scores and baseline scores was 5.6 points ((95% CI: 2.1, 9.0); *p* = 0.002; Cohen’s d = 0.60 (95% CI: 0.22, 0.98)). In the unifocal group this difference was small, only 2.26 points, and not statistically significant ((95% CI: − 1.1, 5.7); *p* = 0.191; Cohen’s d = 0.24 (95% CI: − 0.12, 0.03)). The post-intervention difference in mean MEFS scores between the bifocal group and the unifocal group of 3.3 points ((95% CI: -0.9, 7.5); *p* = 0.120; Cohen’s d = 0.34 (95% CI: − 0.09, 0.77)) had a small effect size and was not statistically significant.

Thus, the effect of treatment on MEFS scores was significant between baseline and post-intervention measurement in the intervention group. However, there was no significant difference in MEFS score between the two intervention groups after the intervention. After the initial improvement with the new optical corrections in the bifocal group, the longitudinal analysis of the MEFS scores showed no significant progression over the one-year follow-up period. Nevertheless, we did obtain some clues that improving near vision may be helpful since the 2.5 dioptres addition for near vision in the bifocals was associated with an average medium-effect improvement of 5.6 points in Total MEFS score with respect to baseline MEFS performance.

#### Cross-sectional relation between visual acuity and the level of post-intervention MEFS scores

At baseline, we found no significant association between visual acuity and MEFS scores^6^. However, at this point in time, most participants were not yet wearing full corrections for their refractive errors, or the correction was out-dated^21^. New prescriptions were given to both groups (bifocal and unifocal) according to the refractive error that was measured at the start of the study. It is therefore of interest to analyze the effect of post-intervention visual acuity on the level of post-intervention MEFS scores. Toward this end, we ran mixed effects models separately for the effects of crowded near visual acuity (n = 68), uncrowded near visual acuity (n = 74) and distant visual acuity (n = 76) on post-intervention T1 and T3 MEFS scores after adjustment of the MEFS scores for gender, calendar age and intervention type (see Methods for details).

**Figure 3:**
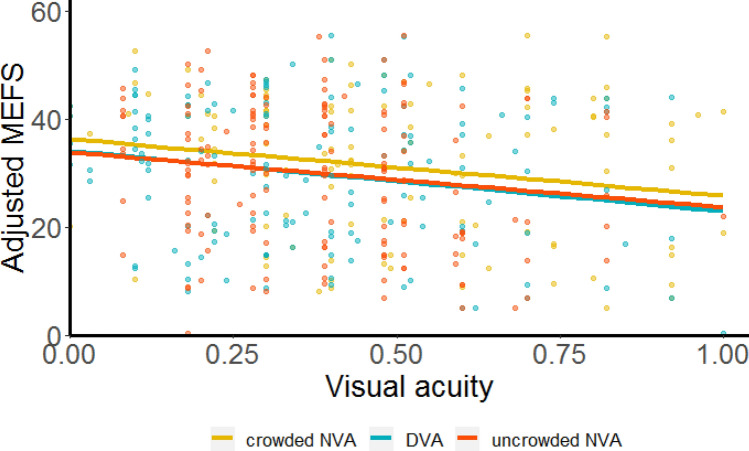
Post-intervention relations between visual acuity and MEFS scores. Post-intervention MEFS scores, adjusted for gender, age and intervention, correlated significantly with each of the three post-intervention visual acuity measures (LogMAR): crowded near visual acuity (orange), uncrowded near visual acuity (red) and distant visual acuity (blue). Individual data points are from T1 and T3. Solid lines are mixed-effects regression lines. Note that better MEFS scores are associated with better post-intervention visual acuities and vice versa.

We found that the crowded near visual acuity, uncrowded near visual acuity and distant visual acuity with newly prescribed glasses were associated significantly with post-intervention adjusted MEFS scores (Fig. 3). The slopes for these relations were of similar magnitude and all negative (Table 1), indicating that after full correction of refractive error, better visual acuity was associated with better MEFS performance. Before pooling of the data across T1 and T3, we verified that there was no statistically significant difference in MEFS scores between T1 and T3.

**Table 1:** Post-intervention relations between visual acuity and MEFS scores.

Cohort's mean visual acuity with newly prescribed glasses	Cohort's mean adjusted MEFS score	Slope
**Crowded near visual acuity**		
0.51 LogMAR	31.0 points (95% CI: 28.1, 33.8)	− 10.5 (95% CI: − 19.8, − 1.3) *p* = 0.025
**Uncrowded near visual acuity**		
0.40 LogMAR	29.8 points (95% CI: 27.1, 32.4)	− 10.2 (95% CI: − 18.6, − 1.8) *p* = 0.019
**Distant visual acuity**		
0.39 LogMAR	29.7 points (95% CI: 27.1, 32.3)	− 11.2 (95% CI; − 22.0, − 0.3) *p* = 0.045

Fixed effects coefficients of the mixed-effects regression analysis. Post-intervention MEFS scores were adjusted for gender, age and intervention, and pooled across T1 and T3.

#### Association between MEFS score changes and changes in ocular alignment

Ocular alignment facilitates visual functions as binocularity (merging the images of both eyes in the brain), accommodation endurance (eyes staying focused at the same near distance for a longer time), stereopsis (depth perception through interpretation of the minimal differences between the image of the right and left eye) and are important in social situations for having eye contact. Possibly, these functions may also play a role in the development of executive functions. We, therefore, analyzed if the change in MEFS scores from baseline to T1 is associated with the change in ocular alignment measured at T1, shortly after the children started wearing the newly prescribed glasses^22^. In this analysis, we found a significant positive rank-correlation between change in ocular alignment and change in MEFS scores in the bifocal group (n = 36, rho = 0.343, *p* = 0.040), indicating that children with improved ocular alignment had improved in MEFS scores. We did not find such a significant rank-correlation in the unifocal group (n = 37, rho = − 0.084, *p* = 0.620), in which the change in strabismus was not statistically significant^22^.

### BRIEF-P and BRIEF questionnaires

In line with our findings at baseline^6^, a year after the intervention, we found no significant correlation between the informant report scores of executive functions and performance of the children on the MEFS test (See supplementary Table S2 online). Unfortunately, all three questionnaires suffered from large percentages of missing scores at both time points, T0 and T3, (missing questionnaires, baseline: BRIEF-P 26%, BRIEF parents’ version 30% and BRIEF teachers’ version 43%. Final assessment: BRIEF-P 47%, BRIEF parents’ version 46% and BRIEF teachers’ version 46%). For this reason, in an attempt to still report on all outcome measures^68^, we only did an exploratory analysis of complete cases on the difference between baseline ratings (T0) and final ratings (T3). Participants with baseline and T3 data on BRIEF-P (bifocals n = 17; unifocals n = 14), or on BRIEF parents’ version (bifocals n = 15; unifocals n = 16) or on BRIEF teachers’ version (bifocals n = 10; unifocals n = 13) were included in these analyses.

#### One year effect of bifocals and unifocals on BRIEF-P and BRIEF scores

After one year, ratings on the teachers’ version of the BRIEF (Global executive composit, GEC) were significantly better than at baseline (change bifocal group: − 21.9 points (95% CI: − 38.3, − 5.5), t(9) = 3.029, *p* = 0.014; Cohen’s d = 1.91 (95% CI: 0.42, 3.41)); change unifocal group: − 16.7 points (95% CI: − 30.6, 2.8), t(12) = 2.625, *p* = 0.022; Cohen’s d = 1.46 (95% CI: 0.23, 2.68). However, this improvement was not significantly different between the two intervention groups (t-test, t(21) = − 0.541, *p* = 0.594; Cohen’s d = 0.23 (95% CI: − 0.60, 1.05)). Scores on the parents ratings BRIEF-P and BRIEF were not significantly different between T0 and T3 (BRIEF-P: t(30) = − 0.937, *p* = 0.336; Cohen’s d = 0.34 (95% CI: − 1.05, 0.37; BRIEF parents version: t(30) = 0.980, *p* = 0.335; Cohen’s d = 0.35 (95% CI: − 1.06, 0.36)). The mean improvement assessed by the teachers was − 19.0 points (95% CI: − 28.7, − 9.2; t(22) = 4.035, *p* = 0.001; Cohen’s d = 1.68 (95% CI: 0.73, 2.63).

For completeness, we checked for differences between the children with completed teachers’ questionnaires at both time points (baseline and final ratings) and those excluded because of missing or incomplete teachers’ questionnaires. The children included in the complete-case analysis tended to have higher adaptive developmental behaviour assessed with the Vineland-S questionnaire compared to the excluded children (49.9 months (95% CI: 45.9, 53.9) and 45.2 months (95% CI: 41.9, 48.5) respectively, t-test, t(53) = 1.772, *p* = 0.082; Cohen’s d = 0.49 (95% CI: − 0.06, 1.04)). There were no differences in age, visual acuities or MEFS scores.

Because of the missingness in both the questionnaires and visual functions and the small number of children with (improved) strabismus we were not able to analyse the associations between parent-rated and teacher-rated executive functions with visual acuities and ocular alignment.

## Discussion

The current multicentre RCT is the first longitudinal study to examine the effect of bifocals and unifocals on task-based executive functions (assessed with MEFS) and rating-based executive functions (assessed with BRIEF-P and BRIEF) in children with DS. The included children were 2 to 16 years old and all received full corrections of their refractive error for distant vision. Although we found no significant differences between the interventions after one year and shortly after the intervention, within the bifocal group there was a change. In this group, the post-intervention MEFS scores were improved significantly compared with the participants’ baseline performance. The effect size of this 1-year change in the bifocal group was medium, whereas the improvement of the unifocal group was statistically small and not significant.

Unfortunately, we could not test reliably if there was an association between improvements in visual acuity and improvements in MEFS scores (Data were too sparse to compute enough pre-post difference pairs). However, our cross-sectional analysis of the post-intervention data showed that better post-intervention MEFS scores were associated with better post-intervention visual acuities (crowded near visual acuity, uncrowded near visual acuity, and distant visual acuity). For participants that had strabismus at baseline, improved ocular alignment with bifocals was associated with improved MEFS scores. Exploratory analysis of the questionnaire data indicated that improvements in executive functioning were also noted by teachers (teachers’ version of BRIEF), with a large effect size, but not by parents (BRIEF-P and parents’ version of BRIEF). After a year, teachers reported fewer problems with executive functioning regardless of the intervention type.

### MEFS

The MEFS is a visual test in which verbal instructions are given, partly supported by visually demonstrated instructions of swiping the picture in the right box. Thus, one might doubt about its value when testing children with low visual acuities. Therefore, we verified the picture size (~ 8 M). The pictures presented in this test are of good contrast and large enough to be easily discriminated by children with limited visual acuity, as poor as 1.5 LogMAR. The mean uncrowded near visual acuity of the participants of our study was 0.58 ± 0.34 LogMAR^21^. None of our participants had an uncrowded near visual acuity poorer than 1.4 LogMAR at baseline. In our previous publication on the baseline measurements of this cohort^6^, we checked the association of the children’s scores with their visual acuity at baseline. We found no statistically significant association. Thus, we could conclude that the MEFS test is suitable for children with visual impairment because the pictures are large enough to be seen by these children, also without optical correction for near vision.

After one year, treatment effects on MEFS scores were not significantly different between the two intervention groups and the longitudinal analysis showed no significant progression over the one-year follow-up period. Perhaps the follow-up period was too short to find statistically significant differences between the two intervention groups. Shortly after participants started wearing their new glasses, near visual acuity (uncrowded and crowded) had improved on average, but it was not until a year later that the effect of bifocals on near vision exceeded the effect of unifocals^21^. Possibly, after a longer follow-up, when the near-vision differences between the intervention groups have developed and had more time to influence the development of executive functions, the better near vision in the bifocal group could lead to a significant difference in the MEFS scores between the intervention groups. Better near visual acuity might also help children with DS to sustainably enhance their visuospatial short-term memory by training, as suggested by other authors^69,70^. However, to study the effects of better near vision on the development of executive functions in children with DS, future studies may need longer follow-up times.

Unfortunately, we could not leverage the longitudinal design of the study to its full potential; there were too many missing baseline measurements of visual acuity (mostly at near) to test if changes in executive functions are directly associated with changes in a child’s visual acuity. Otherwise, we might have been able to account for part of the between-subject variability in the intervention effects. However, we could examine the cross-sectional association between the level of visual acuity and the level of MEFS performance observed after the interventions. We previously found no significant association between baseline visual acuity and baseline MEFS scores^6^. We only found an association between baseline visual acuity and adaptive developmental behaviour (assessed with the Vineland-S questionnaire). However, at baseline, participants were not yet wearing full corrections for their refractive errors. Our finding that better post-intervention visual acuities do correlate significantly with better post intervention MEFS scores is in line with and extends previous cross-sectional studies in visual impaired children without known developmental disorders^23–27^. It also agrees with and extends the findings of Tadic et al.^41^ who compared attentional processes of visual impaired preschool children (without DS and cerebral visual impairment (CVI)) and typically developing children with normal vision. In their cross-sectional study^41^, they reported that visual impairment significantly reduces the capacity of a young child to regulate attention between people and objects, and that in case of visual impairment, the ability to establish attention on toys and maintaining of attention is lower than in children with normal vision.

### Informant reported executive functions

After one year, only teachers reported a substantial improvement of ~ 20 points. This statistically large improvement (Cohen’s d = 1.7) after either intervention represents an ~ 50% reduction of the lag found in the baseline scores of the children with DS relative to age-matched norm scores of typically developing children. At baseline, the mean difference in the teachers’ version of the BRIEF scores compared to the age-matched norm scores was 40.1 points (95% CI: 32.3, 47.9)^6^. Although teachers reported an improvement, parents did not report an improvement in the executive functions of their children with DS. Such a discrepancy between parents’ and teachers’ ratings of behaviour is not uncommon^71,72^. Poor to moderate agreement was observed in children with DS^73,74^, without DS^72,75^, in twins with attention deficit hyperactivity disorder^42^, and analysed in a review already in 1987^71^ and more recently in 2008^43^. Explanations for the discrepancies include the possibility that parents and teachers are observing different behaviours and phenotypes, particularly given the more structured demands at school settings versus less organized home activities, placing different demands on children depending on the setting^42,43^. So, different informants may validly contribute different unique information from different perspectives. Additionally, activities at home may be different from those at school. School activities might include more visually guided activities, which could be more directly influenced and facilitated by better visibility and visual memory support owing to better seeing with new glasses in both intervention groups.

The studies of Daunhauer et al.^76,77^ can also help understand the apparent disagreement between parents and teachers. Their findings include that teachers do encounter the changes in executive functions and are able to rate them in a questionnaire on executive functions. In their cross-sectional study in elementary students with DS, aged 7.86 ± 1.75 years, they demonstrated that executive function skills scored by teachers was the only statistically significant predictor of overall school performance in elementary students with DS^76^. They mention the following two implications. First, executive functions may play a more prominent role in academic contexts for children with DS than was previously noted in literature. Second, their findings suggest that improving executive functions may be of particular use for improving overall school performance in DS. Their findings are an additional motivation to find interventions that can improve executive functions in children with DS. Bifocals with full corrections of refractive errors could be one of them.

### Strengths and limitations

Some of the strengths of the current study are already reported in our previous publications. These include the longitudinal design and the large sample size with a relatively rare biologically well-defined condition (DS). The participants were recruited from rural and urban populations of diverse social status and attended both regular schools and schools for children with special needs, in order to attain a cohort that represents the general Dutch population of children with DS. Further strengths were the multimodal and multi-informant evaluation of intervention efficacy; the robust and standardized measurements that made data collection across multiple sites possible, the use of the combination of both task-based and informant-based measures of executive function differentiating between parent ratings and teacher ratings. The need to obtain reports of both types of observers is highlighted by many authors because of the difference in fundamental behaviours they observe^42,43,75^. Besides the need for different observers in different situations, the combination of task-based assessments (a momentary assessment mostly under optimal conditions) and rating-based assessments (scoring everyday behaviour) is complementary in typically developing children^56,57,62,75^, in preterm preschoolers^58^ and in children with DS^78^. Studies applying such a combination of task-based scores and raters’ information are scant in young children with DS, except for a few studies^78,79^.

Additionally, the current paper focuses on a novel question, i.e., whether visual functions (acuity and ocular alignment) are associated with the level of cognitive performance.

A further strength is the refined visual acuity assessment used in the current study to analyse the association of visual acuity and executive functions, instead of broad visual impairment categories, which were used in previous studies^24,25^, and which do not specify visual acuity at different distances. In our study, we found different timelines for development of uncrowded and crowded near vision and could study the differences between distant and near vision which go unnoticed if these facets of vision are not independently measured. In DS, the difference between distant and near visual acuity is typical if not corrected accordingly, because of their accommodation lag and cerebral visual impairment^21^. Analyses of the correlations with other developmental measures, such as MEFS, were possible because of the refined assessments of visual acuity.

One of the limitations of our study is the limited follow-up time of one year. In children with DS, development is slow. Where a time lapse of one year in typically developing children is often long enough to detect development, in children with DS it may have been too short to detect significant progress in MEFS scores in the unifocal group or a possible difference in MEFS scores between the intervention groups. To reveal differences in slowly developing processes, longer follow-up times are necessary. Possibly, the development of executive functions induced by better visual functions is one of these slow developments, which need time to reach statistically significant differences between baseline and final assessments and between the interventions.

The large age range can also be taken as a limitation. Developmental steps of visual acuity but specially in executive functions are not the same during one year in the youngest ages than in the older ages because of non-linearities in the developmental curve. Especially in children with DS, development is heterogenous. In our study, we included children from the age of two. This is the youngest age at which bifocal use could be expected in the appropriate way. We corrected refractive errors also for near distances in the bifocal group, at the youngest ages possible in order to stimulate the development of visual functions.

The possibility that different teachers might have completed ratings on the same child due to the nature of the trial spanning 12 months might be another limitation. We did not monitor that, because the teachers remained anonymous. Longer follow-up times would have exacerbated this issue even more.

The biggest limitation of our study was the large amount of missing data. For the three versions of BRIEF questionnaires. We tried to deal with this limitation by performing an exploratory analysis of complete cases in order to report the results for all outcome measures. The missing visual acuity data, in particular at baseline, also limited longitudinal analyses. We could not enter all the visual acuity variables in a mixed effects model to analyse the changes in executive functions in relation to changes of visual acuity. We therefore had to limit our analyses to cross-sectional data from the post-intervention visits.

### Overall evaluation of the interventions

After the interventions, the MEFS scores were significantly improved in the bifocal group but not significantly in the unifocal group. Post-intervention, children with better visual functions, crowded and uncrowded near visual acuity and distant visual acuity, showed higher MEFS scores. Children with improvements in ocular alignment typically improved in MEFS scores.

Only explorative analyses could be performed on the BRIEF-P and BRIEF data. Teachers, but not parents, rated improved executive functions in both intervention groups. However, these findings need replication in larger samples with longer follow-up. Such studies could explore if the better post-intervention ratings by teachers and task-based scores on executive functions in DS are a developmental phenomenon or only the result of better visual functioning.

Notwithstanding the acuity improvements as a result of bifocals, children with DS wearing appropriate bifocals still lag behind in visual acuity (far and near) compared to typically developing children^6^.

## Conclusion

After full correction of refractive error, better distant and near vision were associated with higher executive function scores on task-based test administered at near, the MEFS. However, there were not enough data to test such an association with informant reported scores. Nevertheless, teachers’ ratings suggest that at school, children show improved executive functions when wearing full corrections of their refractive error. The +2.5 addition in the bifocals with full correction of refractive error improved near vision more than the full correction of refractive error alone, and bifocals also improved the conditions to achieve better task-based executive function scores on the MEFS.

On the basis of our findings, we suggest to optimize visual functions in children with DS by prescribing them optimal corrections for both distant and near vision to maximize their developmental chances. We found that good corrections for children with DS are up-to-date full corrections of refractive error in bifocals with an addition of +2.5 dioptres for near vision. Further longitudinal research is needed to investigate if improved visual functions indeed boost the development of executive functions in DS.

## Supplementary Information

Supplementary Information.
