# Supplementary material for: One-year effects of bifocal and unifocal glasses on executive functions in children with Down syndrome in a randomized controlled trial
Source: Sci Rep. 2021 Aug 19;11:16893. doi: 10.1038/s41598-021-96308-5 (PMC8377071; doi:10.1038/s41598-021-96308-5)
Supplement: Supplementary file 1 — Supplementary Information. [file 41598_2021_96308_MOESM1_ESM.docx]

Supplementary Materials


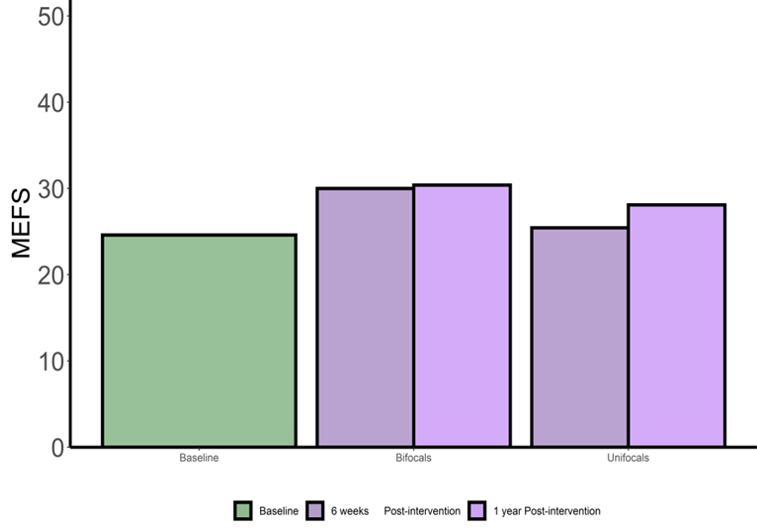


*Figure S1*

*Effect of the interventions on the MEFS controlled for age and gender.*

*Note the significant post-intervention improvement of MEFS Total scores in the bifocal group at T1 (after ~6 weeks) and T3 (after 1-year). The intervention had no significant effect on the MEFS scores in the unifocal group. Post-intervention values were not significantly different between the two groups, as shown in Table S1.*

*Table S1*

*Comparisons op the effects of interventions on MEFS controlled for age and gender.*

*
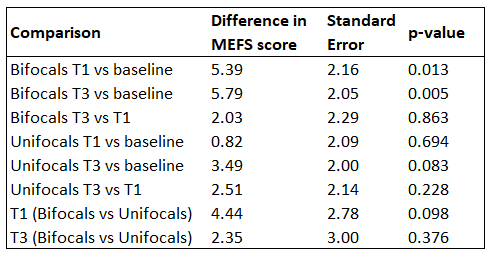
*

*This table lists comparisons of the effects of the interventions on the MEFS Total scores between different time points, baseline, T1 (~6 weeks post-intervention), T3 (final assessment, 1 year post-intervention), as shown in Fig. S1. Note the significant post-intervention improvement of MEFS Total scores in the bifocal group at T1 (after ~6 weeks) and T3 (after 1-year). Within the intervention groups, short-term (T1) and long term (T3) effects were comparable (p>0.1).*

*Table S2*

*Post-intervention rank-correlations*


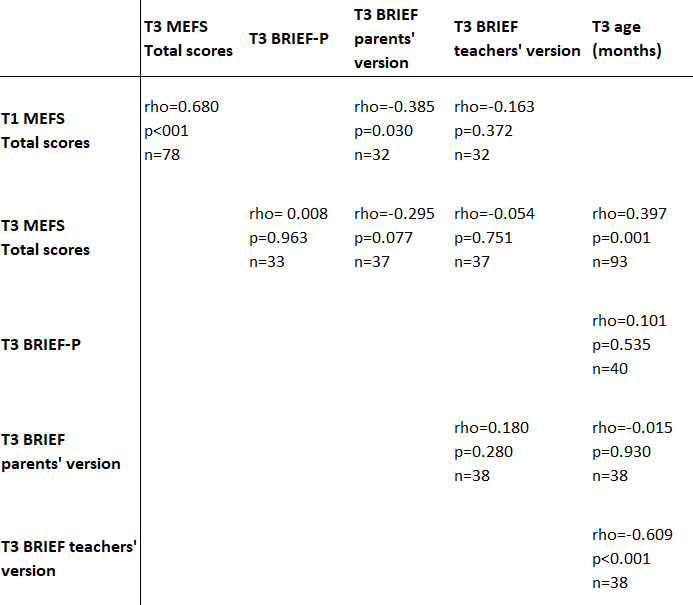


*The table only list rank-correlations for data sets with a sample size of n≥29 participants. Smaller data sets do not have sufficient statistical power to detect even large effect size (rho≥0.50) correlation with Type I and Type II errors of 0.05 and 0.80, respectively [*[*Spearman Correlation: 2-tailed (statisticssolutions.com)*](https://urldefense.com/v3/__https:/www.statisticssolutions.com/wp-content/uploads/wp-post-to-pdf-enhanced-cache/1/spearman-correlation-2-tailed.pdf__;!!HJOPV4FYYWzcc1jazlU!u5LY7RpwKCR4iHxoWbixE6JOX--BRVQbjgS1BO18n408s-WadRRAuFB-1WpGM7cyvbt9$)*].* *Note, that this table should be interpreted with caution since each correlation measure is derived from a different subgroup of children.*
